# Remote Sensing of Soil Water Retention Signatures Using Sentinel-2 Time-Series and Exponential Decay Fitting Model

**DOI:** 10.3390/s26123709

**Published:** 2026-06-10

**Authors:** Linghua Meng, Ya Chen, Shinai Ma, Yihao Wang, Huanjun Liu

**Affiliations:** 1 State Key Laboratory of Black Soils Conservation and Utilization, Northeast Institute of Geography and Agroecology, Chinese Academy of Sciences, Changchun 130102, China; menglinghua@iga.ac.cn (L.M.); mashinai@iga.ac.cn (S.M.); wangyihao@iga.ac.cn (Y.W.); liuhuanjun@iga.ac.cn (H.L.); 2 Youyi Observation and Research Station of Modern Agriculture, Northeast Institute of Geography and Agroecology, Chinese Academy of Sciences, Shuangyashan 155100, China; 3 School of Public Administration and Law, Northeast Agricultural University, Harbin 150030, China

**Keywords:** soil water retention capacity (SWRC), soil moisture decay index (SMDI), time-series NDWI, exponential decay fitting model (EDFM)

## Abstract

**Highlights:**

**What are the main findings?**

- Time-series NDWI exhibited distinct exponential decay signatures that vary significantly across soil textures and degradation gradients.
- SMDI showed high consistency with in situ soil moisture and significant positive correlations with SOM and FC.

**What are the implications of the main findings?**

- This study successfully transformed point-scale static SWRC measurements into spatially continuous monitoring data at 10-m resolution.
- SMDI spatial mapping accurately identifies impaired water-holding functions, providing digital decision-making support for precision black soil conservation and fertility restoration.

**Abstract:**

Soil water retention capacity (SWRC) is vital for agriculture and watersheds, but traditional measurements are hindered by destructive sampling and spatial discontinuity. This study selected Youyi and Heshan farm in Heilongjiang Province as the study area, using the time-series Normalized Difference Water Index (NDWI) from Sentinel-2 during the snowmelt-to-bare-soil window as a soil water retention signature (SWRS) for monitoring SWRC. The exponential decay fitting model (EDFM) was used to construct a Soil Moisture Decay Index (SMDI) to analyze the spatial patterns of the SWRC. Results showed that: (1) time-series NDWI exhibited distinct exponential decay signatures varying with soil textures and degradation gradients; (2) the EDFM effectively fitted the time-series NDWI (R^2^ = 0.84–0.99), extracting decay rate and stable level to quantify SWRC; (3) SMDI showed high consistency with in situ soil moisture (R = 0.82–0.88) and measured field capacity (Youyi Farm: R^2^ = 0.56; Heshan Farm: R^2^ = 0.59), and correlated significantly with soil organic matter (R^2^ = 0.61–0.71) and texture (R^2^ =0.50–0.64), confirming the physical controls on water retention; and (4) SMDI spatial distribution revealed distinct degradation patterns across varying topographic and soil conditions. This study innovatively transformed point-scale static SWRC measurements into spatially continuous monitoring, offering new tools for precision water management and degraded-soil restoration, with strong theoretical and practical value.

## 1. Introduction

Soil Water Retention Capacity (SWRC) denotes an inherent soil hydraulic characteristic that essentially regulates agricultural productivity, ecosystem stability, and watershed hydrological regulation [1]. Intensive agricultural exploitation has resulted in severe soil degradation, manifested as physical compaction and the depletion of organic matter, which fundamentally modifies the soil’s pore structure in the black soil regions. Therefore, there is an immediate necessity for high-precision monitoring of SWRC to precisely diagnose these hydrological changes and implement targeted restoration measures for the conservation of black soils.

Traditional methods quantify SWRC through the soil water retention curves, which describe the functional relationship between soil water content and matric potential [2]. These curves are typically measured at the point scales using laboratory instruments such as pressure plates and tensiometers [3], or estimated through pedotransfer functions based on soil texture and organic matter [4]. Although these methods provide high-precision measurements, they are severely constrained by destructive sampling, prohibitive costs, and limited spatial continuity [2]. Consequently, remote sensing has become an essential tool for soil moisture monitoring due to its capacity for large-scale, synchronized, and all-weather observations [5,6]. In this context, ground-based, proximal, and satellite remote sensing of soil moisture have achieved high-confidence retrievals of surface soil water content through the precise capture of land-surface electromagnetic signals [6]. Specifically, optical remote sensing (such as Sentinel-2 and Landsat) achieves spatial resolutions of 10–30 m, and it can detect instantaneous surface moisture [7], but it remains incapable of resolving natural decay trajectories of soil water content. Synthetic Aperture Radar (SAR) technology (such as Sentinel-1) provides all-weather monitoring capabilities at 10–20 m resolution [8]; however, it similarly retrieves only instantaneous surface moisture and suffers from significant interference caused by vegetation and surface roughness [9]. Furthermore, while passive microwave sensors at the L-band (e.g., SMAP, SMOS) offer improved vegetation penetration compared to C-band SAR, retrieving soil moisture during the crop growing season remains challenging due to complex vegetation scattering and emission interactions [10,11]. Ultimately, while remote sensing technologies enable global soil moisture monitoring, existing products predominantly capture instantaneous surface states rather than the intrinsic stabilization dynamics characterizing the SWRC [12].

Meanwhile, the capacity of remote sensing time-series analysis to capture continuous dynamic variations has led to its extensive application in soil moisture research. The use of time-series remote sensing data enables the detection of abnormal fluctuations in soil moisture, thereby facilitating the quantitative assessment of regional drought events throughout their entire lifecycle—from onset and progression to recovery. Similarly, time-series analysis of the Normalized Difference Water Index (*NDWI*) has been extensively used to characterize surface wetness dynamics. Li, et al. [13] confirmed that Sentinel-2 time series can effectively capture surface soil moisture dynamics during freeze–thaw periods. Furthermore, studies using SMAP satellite time series have constructed global drydown rate datasets, revealing significant disparities in water retention capacity across diverse climatic zones and soil types [14]. The effectiveness of multi-source remote sensing in resolving seasonal decay patterns has been validated through the analysis of dynamic moisture mechanisms across various ecological regions [15]. In particular, the temporal evolution of these drydown processes has been found to conform to exponential decay laws, where the characteristic decay time quantifies the ‘memory’ duration and release rate of soil water [16]. Despite these advancements, mapping the fine-scale spatiotemporal distribution of the SWRC remains challenging.

However, remote sensing time-series data are frequently obscured by cloud cover, atmospheric disturbances, and surface heterogeneity, resulting in discontinuous, noisy, or non-smooth profiles [17], necessitating mathematical model fitting to extract reliable physical parameters. Compared with simple linear regression, which inadequately characterizes decay rate variations, the Exponential Decay Fitting Model (EDFM) strikes a balance between physical mechanisms and computational efficiency. McColl, et al. [18] verified that exponential decay time scales reflect soil moisture release rates across contrasting soil textures, while Lehmann and Or [19] demonstrated that these water release rates are fundamentally governed by soil texture and hydraulic properties. Requiring only time-series observations [20], the EDFM establishes a mathematical foundation for retrieving intrinsic soil water retention characteristics through high-resolution remote sensing time series. Nevertheless, its applicability to high-resolution sequences in complex, heterogeneous black soil regions remains to be explored. Whether it can effectively decouple the intrinsic SWRC from surface noise and serve as a reliable proxy for quantifying land degradation remains a critical question, necessitating further empirical validation across diverse soil textures and degradation gradients.

The black soil region in Northeast China (NEC), representing one of the four largest Mollisol zones globally, is experiencing severe degradation characterized by topsoil thinning and accelerated erosion, particularly in undulating landscapes [21,22]. Although extensive studies have advanced the inversion of soil organic matter and texture through machine learning algorithms [23,24], they have further highlighted the critical role of bare-soil windows in improving remote sensing mapping accuracy for black soil properties [25]. Nevertheless, transitioning from the static mapping of soil properties to the dynamic characterization of the SWRC remains a significant challenge that has been largely overlooked in current remote sensing research. Given that SWRC serves as a critical buffer for sustaining crop yields and mitigating moisture stress, quantifying its dynamics is vital for evaluating the functional consequences of black soil degradation. Therefore, to address these problems, this study selected the agricultural reclamation areas of Youyi and Heshan Farms in Heilongjiang Province as study areas, aiming to develop a novel remote sensing framework to map and quantify the spatiotemporal distribution of the SWRC.

The specific objectives are as follows: (1) to analyze time-series *NDWI* and characteristic changes across degradation gradients, (2) to determine the applicability of exponential decay fitting model in time series, (3) to construct a soil moisture decay index (*SMDI*) to analyze the spatial patterns of the SWRC, and (4) to compare the SWRC differences in different soil degradation among the study area and discuss the corresponding variation patterns, genesis, and insights.

## 2. Materials and Methods

### 2.1. Study Area

**Figure 1:**
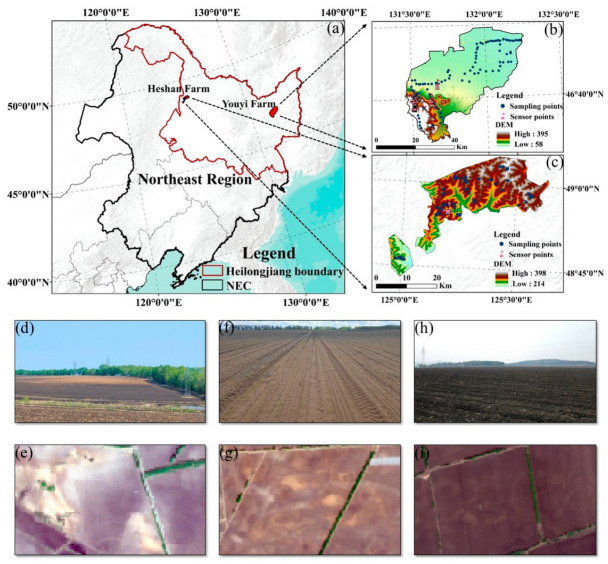
Overview map of the study area: (**a**) is the location of the study area, (**b**,**c**) are DEMs of Youyi Farm and Heshan Farm, (**d**,**e**) are site photos and true-color images of heavily degraded black soil acquired on 8 May 2024 (black soil layer: 0 < h ≤ 20 cm), (**f**,**g**) are site photos and true-color images of degraded sandy black soil acquired on 8 May 2024, and (**h**,**i**) are site photos and true-color images of degraded loamy soil acquired on 8 May 2024.

For this study, Youyi Farm (131°29′–132°15′ E, 46°28′–46°59′ N) and Heshan Farm (129°30′–130°10′ E, 47°10′–47°35′ N) in Heilongjiang Province, China were selected as the study areas (Figure 1a). Both farms share a temperate continental monsoon climate. Mean annual temperatures are approximately 2.5 °C and 3.2 °C, respectively, with spring (March–May) mean temperatures ranging from 2.8 °C to 6.5 °C. The mean annual precipitation is 550–600 mm and 500–550 mm, respectively, of which 75% concentrates in the growing season from June to September [26,27]. Elevations at Youyi Farm range from 58 m to 395 m (Figure 1b), gradually decreasing from west to east. Conversely, elevations at Heshan Farm range from 214 m to 398 m (Figure 1c), exhibiting an opposite topographic pattern (higher in the east and lower in the west) characterized by prevalent sloping farmlands. The study area is dominated by Mollisols (black soils), with local sandy patches occurring in severely degraded areas [22,23]. The terrain consists primarily of undulating slopes (0–6°) with intermittent low ridges and shallow valleys. No bedrock outcrops occur within the cultivated layer; however, residual rock fragments are sporadically exposed in severely eroded slope shoulders. Long-term agricultural reclamation and intensive mechanized farming have substantially altered the soil physical properties, resulting in severe black soil degradation (Figure 1d–i). Ridge formation is conducted in October, following the crop harvest, after which the farmland is left fallow and completely covered by snow throughout the winter. This agricultural practice ensures a bare-soil condition without vegetation interference upon spring snowmelt, providing an optimal window for optical remote sensing of soil water retention signatures (SWRS). Affiliated with the Beidahuang Group, these two study areas serve as strategic benchmarks for a region-wide management system covering 113 state farms (3.25 × 10^6^ ha). The consistency of farming protocols across this vast reclamation area ensures that the remote sensing models developed in this study possess robust scalability, offering a ready-to-use template for operational soil degradation monitoring at a regional scale.

### 2.2. Data Acquisition and Preprocessing

#### 2.2.1. Remote Sensing Data

Sentinel-2 Level-2A data were acquired from the European Space Agency in 2024, covering the snowmelt-to-bare-soil period (March–May). The images were pre-processed with ortho-correction, geometric fine correction, and atmospheric correction. Cloudy scenes were excluded using the Sentinel-2 QA60 quality flag. Residual outliers in the *NDWI* time series caused by thin clouds or snow were identified using threshold filtering and replaced with linear interpolation between adjacent valid dates.

#### 2.2.2. Other Data

DEM data were obtained from the ALOS World 3D 30 m (AW3D30) dataset. This study determined different slope positions by extracting slopes using DEM data.

Farmland boundary vector data and crop type data were provided by the farms.

MODIS snow product data were derived from the Terra and Aqua satellites; common products included MOD10A1 and MYD10A1, which provided global daily snow cover data at 500 m resolution. These data confirmed that snowmelt typically begins in early March, guiding the 2024 study window.

#### 2.2.3. Soil Sample Data

In this study, 21 in situ soil temperature and moisture sensors were deployed at a 10 cm depth within the 0–10 cm topsoil layer in June–September 2024 to monitor surface soil water content changes. The sensors were placed across the full range of field conditions, covering non-degraded (ND), lightly degraded (LD), moderately degraded (MD), and heavily degraded (HD) soils, as well as low-slope (LS), moderate-slope (MS), and high-slope positions.

Field capacity (FC) was measured at 71 sampling positions at Youyi Farm and 31 at Heshan Farm in 2024, to provide independent validation data. In addition, 98 soil samples from Youyi Farm and 130 soil samples from Heshan Farm were collected in April–May 2024. The soil samples were air-dried and ground, then analyzed for soil organic matter (SOM) and soil texture in the laboratory. These sensors were deployed from June to September because spring freeze–thaw cycles prevent installation and damage equipment. Summer rainfall provided saturated conditions for observing decay. The post-rainfall decay patterns were used to validate the spring NDWI decay characteristics, because both reflect the intrinsic soil pore structure that governs SWRC.

### 2.3. Time-Series NDWI Analysis

In this study, the Normalized Difference Water Index (*NDWI*) was calculated using Green (Band 3) and NIR (Band 8) channels following [28]. Although SWIR-based *NDWI* is widely used for vegetation or urban water mapping [29], this NIR–SWIR combination is also termed the Modified Normalized Difference Water Index (MNDWI) [30]. During the initial method development stage, we compared this NIR–SWIR MNDWI against Green–NIR *NDWI* over the same bare-soil window. The Green–NIR combination was preferred here for three reasons: (1) its superior spatial resolution (10 m vs. 20 m); (2) its reduced susceptibility to mineral interference in bare-soil conditions, which often affects SWIR bands in complex soil environments; and (3) *NDWI* exhibited clearer separation among different soil textures and slope positions than MNDWI.

(1) $$\mathrm{NDWI}=\frac{\rho_{\mathrm{Green}}-\rho_{\mathrm{NIR}}}{\rho_{\mathrm{Green}}+\rho_{\mathrm{NIR}}}$$

**Figure 2:**
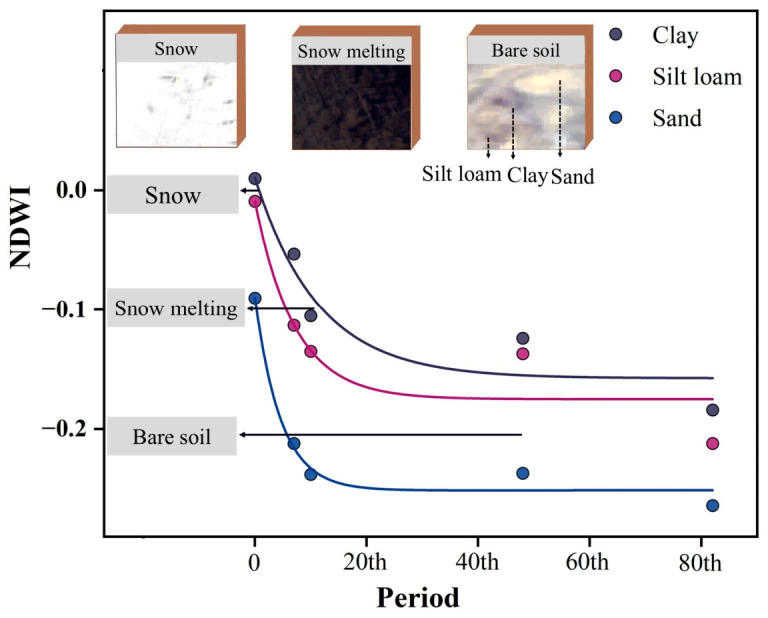
Time-series NDWI in different soil textures from snowmelt-to-bare-soil period (the date of complete snowmelt as the temporal origin = 0).

We extracted time-series *NDWI* to construct time-series curves extending through the bare-soil period from snowmelt (Figure 2). The soil moisture exhibited a progressive decline driven by natural evaporation, stabilizing approximately 15–20 days after snowmelt. This period represents the natural attenuation cycle from saturation to stability across all observed soil types, and it was used as the analytical window for subsequent model fitting. This time series presents the complete transition from saturated to stable states. Comparisons among soil textures revealed that clay exhibits the slowest decline with the highest stable value, sand shows the most rapid decline with the lowest stable value, and silt loam falls between these extremes. These differences arise from textural variations in soil pore structure: clay is rich in micropores with strong capillary action, whereas sand is dominated by macropores that facilitate rapid drainage [31]. These distinct decay characteristics provide a physical basis for subsequent quantitative parameter extraction.

### 2.4. Exponential Decay Fitting Model (EDFM) and SMDI Construction

The EDFM was widely employed to characterize natural attenuation processes in physical systems [32]. In this study, time-series *NDWI* from snowmelt showed clear exponential decay curves (Figure 2). This pattern aligns with the EDFM framework, allowing direct fitting of the model to time-series *NDWI*. The formula (Equation (2)) is as follows:

(2) $${NDWI}_{\left(t_{i}\right)}=\;a\times exp(-t_{i}/b)+c$$

where $t_{i}$ represents the *i*-th day since complete snowmelt; a represents the *NDWI* value immediately after complete snowmelt; b represents the characteristic decay time, characterizing soil water release resistance; and c represents the stable asymptote, representing the stable water retention level.

The in situ soil moisture sensor data were also processed using the same EDFM approach. This processing methodology maintains consistency with the time-series *NDWI* fitting logic, thereby ensuring that decay characteristics derived from both data sources share a unified physical interpretation and providing a methodological basis for subsequent validation.

**Figure 3:**
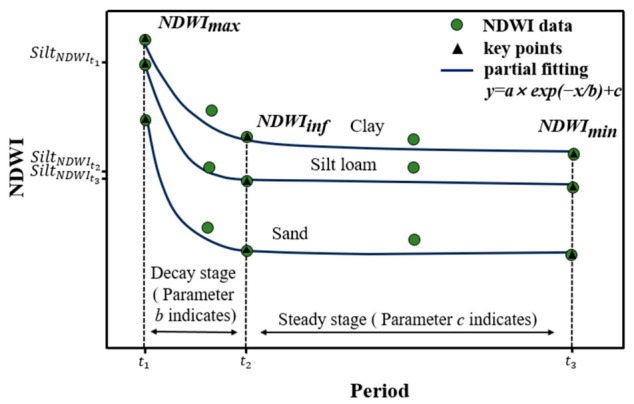
SMDI construction from characteristic parameters of time-series NDWI based on EDFM.

To comprehensively characterize SWRC, the Soil Moisture Decay Index (*SMDI*) was constructed by combining these parameters with fitted *NDWI* values at three critical temporal stages: the onset of decline, the cessation of decline, and the stabilization point (Figure 3). *SMDI* integrates the soil water release resistance during the decay stage and the equilibrium retention level at the steady stage into a single metric, where a higher *SMDI* value indicates stronger soil water retention capacity and vice versa. Equation (3) is as follows:

(3) $$SMDI={(NDWI}_{t_{1}}+{NDWI}_{t_{2}})\times b+{(NDWI}_{t_{2}}+{NDWI}_{t_{3}})\times c$$

where $t_{1}$ is the day of complete snowmelt (*t* = 0), identified from MODIS snow products, when soil surface water begins to decrease; $t_{2}$ is the time when the fitted *NDWI* curve ceases to decline, marking the end of rapid water loss; $t_{3}$ is the time when the fitted *NDWI* reaches the asymptote *c*, indicating the equilibrium state; ${NDWI}_{t_{1}}$ is the fitted value of *NDWI* at $t_{1}$; ${NDWI}_{t_{2}}$ is the fitted value of *NDWI* at $t_{2}$; ${NDWI}_{t_{3}}$ is the fitted value of *NDWI* at $t_{3}$; b is the fitted decay constant for each pixel, and prior to *SMDI* calculation, it was normalized to [−1, 1] per pixel to ensure dimensional consistency; and c is the fitted stable asymptote (equilibrium *NDWI* value) for each pixel.

### 2.5. Model Accuracy Validation and Evaluation

In this study, independent-sample validation was comprehensively employed to assess the accuracy of the *SMDI* through multi-dimensional field measurements and intrinsic soil property analyses. *SMDI* was first validated against continuous in situ soil moisture dynamics from independent sensor networks deployed across diverse soil textures and degradation gradients (17 sensors at Youyi Farm and 4 sensors at Heshan Farm). Additionally, the *SMDI* was cross-validated against measured FC at independent sampling points to confirm its ability to represent the SWRC. Furthermore, correlations between *SMDI* and key soil physicochemical properties, including SOM and texture composition (sand, silt, and clay), were thoroughly analyzed to establish its physical basis. All model performance metrics were quantified using the correlation coefficient (R), coefficient of determination (R^2^), and root-mean-square error (RMSE) [7,33], with R^2^ also serving to assess the accuracy of the exponential decay model.

## 3. Results

### 3.1. Time-Series NDWI Responses Across Different Degradation and Model Fitting

**Figure 4:**
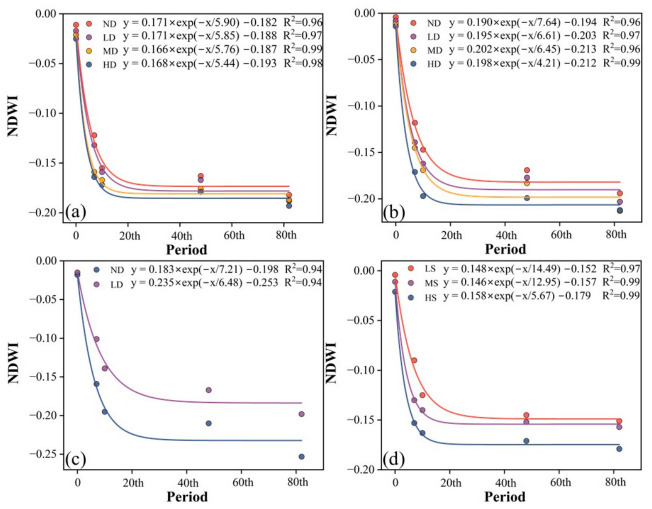
Time-series NDWI across degradation gradients and model fitting: (**a**) black soil (thickness > 20 cm), (**b**) black soil (0 < thickness ≤ 20 cm), (**c**) sandy soil, and (**d**) loamy soil. ND represents non-degraded, LD represents lightly degraded, MD represents moderately degraded, and HD represents heavily degraded; LS represents low slope, MS represents moderate slope, and HS represents high slope.

Before formal model fitting, Green–NIR *NDWI* and NIR–SWIR MNDWI were intercompared over the identical bare-soil window. *NDWI* captured texture-dependent and topographically controlled decay differences more distinctly than MNDWI, thereby supporting its exclusive use in subsequent analyses (Detailed time-series data in Figures S1–S4). Figure 4 shows the time-series *NDWI* variations across different soil textures, degradation levels, and different slope positions, with all curves fitted by EDFM with R^2^ exceeding 0.94. For black soil thicknesses greater than 20 cm (Figure 4a), the decay time constant *b* decreased from 5.90 under non-degraded (ND) conditions to 5.44 under heavy degradation (HD), while stabilization level *c* dropped from −0.182 to −0.193, indicating that heavily degraded black soils lose moisture more rapidly and exhibit lower water-retention capacity. A consistent trend was observed for soils with a thickness between 0 and 20 cm (Figure 4b), where *b* fell from 7.64 (ND) to 4.21 (HD), and *c* declined from −0.194 to −0.212. Sandy soils (Figure 4c) also exhibited a faster decay under low degradation (LD) conditions, with *b* decreasing from 7.21 (ND) to 6.48 (LD), and the stabilization level dropping from −0.198 to −0.253. For loamy soils (Figure 4d), slope position exerted a strong control: *b* decreased sharply from 14.49 on the lower slope (LS) to 5.67 on the high slope (HS), while *c* fell from −0.152 to −0.179. Overall, all curves followed a similar two-stage pattern, with a rapid decline followed by stabilization, but the decay rate and stable level differed clearly among different degradation states, slope positions, and soil textures. Specifically, HD and HS soils exhibited smaller *b* values and lower *c* values, signifying a weakened SWRC. Sandy soils followed the same trend relative to loamy and clay soils. Thus, *b* and *c* jointly reflect how degradation, slope, and texture control water retention. Bootstrap resampling of 2000 randomly selected pixels further confirmed that the fitted parameters were robustly constrained (median R^2^ = 0.908), with detailed uncertainty statistics provided in Table S1.

### 3.2. Field Validation of SMDI Estimates

**Figure 5:**
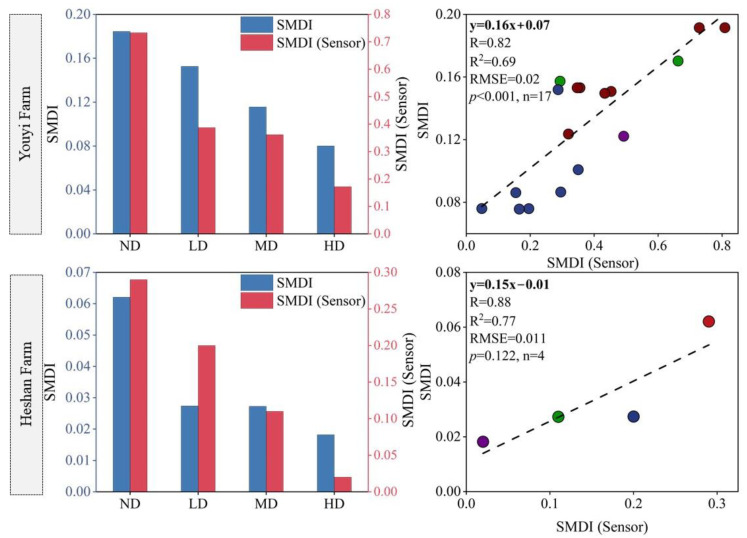
Field validation of SMDI against in situ soil moisture sensors at Youyi Farm and Heshan Farm.

**Figure 6:**
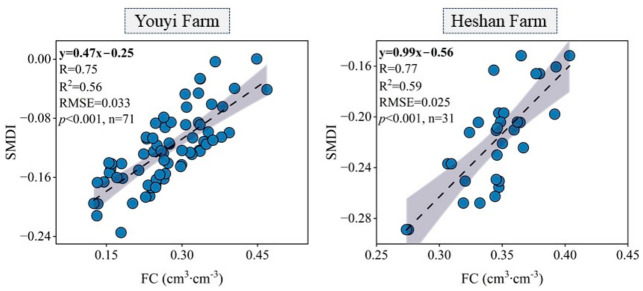
Relationship between SMDI and measured FC at Youyi Farm and Heshan Farm.

As illustrated in Figure 5, independent validation using in situ soil moisture sensors (0–10 cm depth) revealed strong agreement between the *SMDI* estimates and the sensor-derived *SMDI* (*SMDI*(Sensor)) at both farms, with correlation coefficients of 0.82 at Youyi Farm and 0.88 at Heshan Farm. This high consistency demonstrated that the remote sensing-derived decay parameters accurately capture actual soil moisture depletion rates from saturation to stability. Additionally, as shown in Figure 6, *SMDI* exhibited a significant positive correlation with laboratory-measured FC at Youyi Farm (R = 0.75, R^2^ = 0.56, RMSE = 0.033, *p* < 0.001, n = 71) and Heshan Farm (R = 0.77, R^2^ = 0.59, RMSE = 0.025, *p* < 0.001, n = 31), further substantiating that the index effectively quantifies intrinsic SWRC. The robust performance across independent sensors and measured FC validates that the EDFM-fitted *NDWI* decay trajectories could effectively capture the SWRC, confirming that optical remote sensing is capable of characterizing the SWRC through time-series decay patterns rather than merely capturing instantaneous moisture states.

### 3.3. Relationships Between SMDI and Soil Properties

**Figure 7:**
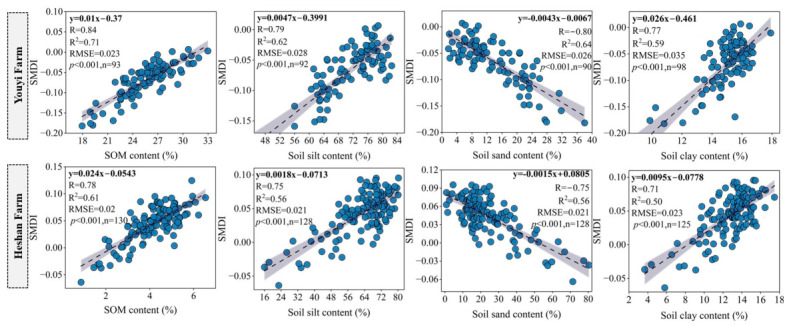
Scatter plot of soil properties and SMDI.

*SMDI* exhibited significant correlations with intrinsic soil physicochemical properties across both farms (Figure 7). At Youyi Farm, *SMDI* increased markedly with SOM content (R = 0.84, R^2^ = 0.71), while similar positive trends were observed at Heshan Farm (R = 0.78, R^2^ = 0.61). Regarding soil texture, *SMDI* showed opposing responses to particle fractions. Sand content was negatively correlated with *SMDI* at both sites (Youyi: R = −0.79, R^2^ = 0.62; Heshan: R = −0.75, R^2^ = 0.56), indicating rapid drainage through macropores. Conversely, silt contents exhibited significant positive correlations (Youyi: R = 0.79, R^2^ = 0.62; Heshan: R = 0.75, R^2^ = 0.56), as did clay contents (Youyi: R = 0.77, R^2^ = 0.59; Heshan: R = 0.71, R^2^ = 0.50), demonstrating the dominance of micropore-driven water retention. These robust correlations confirm that SWRC is intrinsically governed by SOM and texture, validating that *SMDI* effectively captures the soil physical controls on water retention.

### 3.4. Spatial Distribution of SWRC Based on SMDI

**Figure 8:**
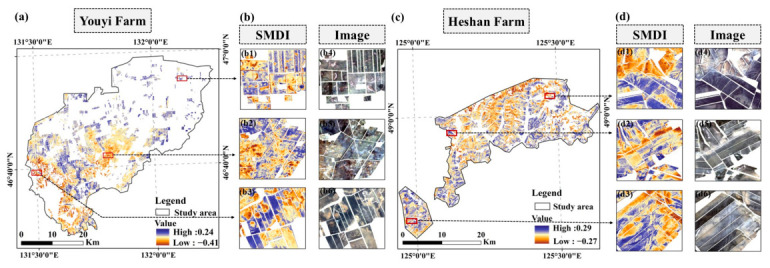
Spatial distribution of SWRC derived from SMDI. (**a**) Youyi Farm. (**b**) Representative areas at Youyi Farm: ((**b1**–**b3**)) magnified SMDI results and ((**b4**–**b6**)) corresponding Sentinel-2 true-color composites. (**c**) Heshan Farm. (**d**) Representative areas at Heshan Farm: ((**d1**–**d3**)) magnified SMDI results and ((**d4**–**d6**)) corresponding Sentinel-2 true-color composites.

The spatial distribution of the SWRC derived from the *SMDI* revealed distinct regional patterns across both farms (Figure 8). At Youyi Farm (Figure 8a), the *SMDI* exhibited a pronounced east–west gradient, with higher values concentrated in the east and lower values dominating the central and western areas. The western zone, characterized by rugged topography and severe soil degradation, displayed a markedly weakened SWRC, while the central plains showed a reduced *SMDI* corresponding to extensive sandy soils with intrinsically poor water retention. In contrast, Heshan Farm demonstrated an inverse spatial layout (Figure 8c), with strong SWRC in the western region and weak SWRC in the central and eastern sectors. The eastern and central areas exhibited significantly depressed *SMDI* values, which are attributable to steep slopes, severe soil erosion, and extensive black soil degradation, whereas the western region maintained a higher SWRC associated with gentler terrain and less degraded conditions. Detailed zoomed views (Figure 8b,d) further validated these spatial heterogeneities at the field scale.

## 4. Discussion

### 4.1. Performance of Time-Series NDWI Under Different Degradation Conditions

This study recorded soil moisture attenuation dynamics under varying textures and degradation levels during the bare-soil window following snowmelt using time-series *NDWI* and obtained spatial distribution results of the SWRC at 10 m resolution. Figure 4 shows that the R^2^ value of EDFM’s fitting accuracy for the time-series *NDWI* curves exceeds 0.94. The attenuation characteristics vary significantly across different soil textures, originating from distinct soil pore structures: loam and black soil contain higher clay and silt content and exhibit abundant micropores and strong capillary action, thereby leading to a slow water release, whereas sandy soil is dominated by macropores, features efficient drainage, and has a weak SWRC [34]. The study by Cosby, et al. [35] also confirmed that soil pore structure governs water retention and release, thereby determining the SWRC of different soil textures. Furthermore, this study demonstrated that within the same soil texture, varying degrees of degradation significantly altered the attenuation characteristics. For instance, under ND conditions with a black soil layer thickness exceeding 20 cm, the parameter b was 5.90 and c was −0.182, whereas under HD conditions, b decreased to 5.44 and c dropped to −0.193, indicating accelerated water loss and a reduced SWRC. For black soils with a thickness of 0–20 cm, this difference was more pronounced: the b value of ND was 7.64, while that of HD dropped sharply to 4.21, and the c value decreased from −0.194 to −0.212. This phenomenon is attributed to degradation-induced soil organic matter (SOM) depletion and soil structure disruption, which lead to a reduction in micropores and an increase in macropores, thereby facilitating accelerated water loss [36]. This finding was also corroborated by previous studies demonstrating that changes in soil texture and organic matter significantly altered the SWRC [4,37]. Notably, research on farmland in the NEC black soil region indicates that erosion markedly altered soil physical properties, with field capacity declining substantially as erosion intensity increases [22].

**Figure 9:**
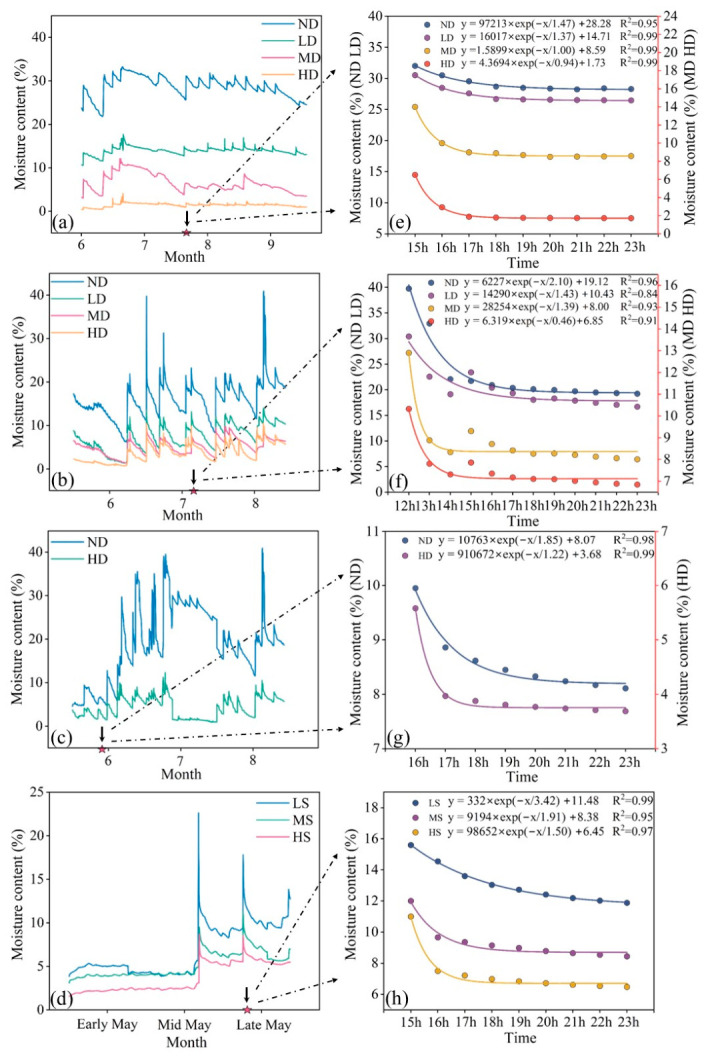
Time-series soil moisture with different degradation gradients and model fitting. ((**a**–**d**)) In situ soil moisture dynamics from May to September 2024: (**a**) black soil (h > 20 cm), (**b**) black soil (0 < h ≤ 20 cm), (**c**) sandy soil, and (**d**) loamy soil. (**e**–**h**) Corresponding EDFM fittings of post-rainfall soil moisture decay curves for (**a**–**d**), respectively.

Meanwhile, to verify whether the time-series *NDWI* could accurately reflect soil moisture changes, this study compared the results with data from in situ soil temperature and moisture sensors. Notably, due to the alternating freeze–thaw cycles in spring, sensors could not be deployed during the initial snowmelt period. Therefore, the instrumentation was installed under normal soil conditions during relatively concentrated rainfall periods from May to September. The change in soil moisture from saturation to stabilization was observed during typical rainfall events, thereby simulating the natural decay process of soil moisture analogous to the extended post-snowmelt drawdown. Because the decay pattern is inherently dictated by the intrinsic soil pore structure, this method is not limited to the spring season; rather, any temporal window exhibiting a complete saturation-to-stabilization drydown process can effectively capture the SWRC. Figure 9e–h demonstrated that the R^2^ values for EDFM’s fitting of soil moisture curves all exceed 0.84. During post-rainfall attenuation, HD soil moisture decreased significantly faster than ND, HS decreased faster than LS, and sandy soil exhibited a faster attenuation rate than black soil and loam. This aligned with the pattern observed in the time-series *NDWI* in Figure 4, confirming that the attenuation signals captured by remote sensing genuinely reflect actual soil moisture changes. This physical behavior concurs with the findings of Shellito, et al. [38] in their study conducted on the continental United States, demonstrating that the drying rate of bare soil is governed by soil properties and follows an exponential decay pattern. However, current research on SWRC has primarily relied on point-scale station records or coarse-resolution gridded products, with spatial resolutions typically exceeding 1 km [39]. These methods can only reveal regional trends and fail to distinguish SWRC variations within individual plots caused by erosion or degradation [40]. In contrast, this study applied the EDFM to 10 m resolution Sentinel-2 optical imagery, maintaining high fitting reliability across the black soil region. The resulting time-series *NDWI* trends were successfully validated through the field sensors, demonstrating that the exponential attenuation law is applicable not only to high-frequency continuous data but also enables the extraction of the soil’s intrinsic SWRC from relatively sparse high-resolution optical remote sensing imagery.

### 4.2. Advantages of the SMDI for SWRC Monitoring

Both the SWRC and FC vary considerably across fields due to the coupled influences of topography and soil properties [41]; consequently, spatially isolated point measurements frequently fail to capture regional-scale heterogeneities. In addition, traditional measurements rely on destructive sampling at discrete locations; mapping full-field spatial variations remains difficult. Concurrently, existing satellite-derived soil moisture products face a comparable limitation: each pixel blends electromagnetic signals radiating from soil texture, vegetation, and terrain, thereby complicating the direct translation of these products into field-scale hydraulic parameters [42]. Although recent advances have leveraged machine learning to estimate water retention curves from basic soil properties [43], these methods remain dependent on spatially dense ground data for training and execution. In this study, the strong correlation between the *SMDI* and the measured FC demonstrates that the soil water retention signatures captured via post-snowmelt or post-rainfall optical time series can serve as an effective alternative to dense field sampling while faithfully capturing the SWRC. This diagnostic capability is physically justifiable: SOM improves the pore structure and capillary forces, while soil texture controls water drainage rates by defining the pore-size distribution [35,44]. Our results show *SMDI* increased with SOM, clay, and silt, and decreased with sand (Figure 7), closely aligning with these foundational soil physics principles.

**Figure 10:**
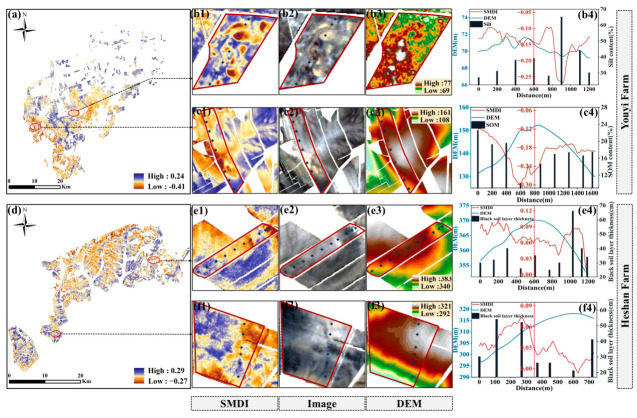
Spatial distribution of SMDI in typical degraded plots. (**a**) SMDI spatial distribution at Youyi Farm. (**b1**) and (**c1**) are magnified SMDI maps of two typical degraded plots at Youyi Farm; (**b2**)/(**c2**), (**b3**)/(**c3**) and (**b4**/(**c4**) are the corresponding true-color images, DEMs, and profiles of SMDI, DEM and soil properties, respectively. (**d**) SMDI spatial distribution at Heshan Farm. (**e1**) and (**f1**) are magnified SMDI maps of two typical degraded plots at Heshan Farm; (**e2**)/(**f2**), (**e3**)/(**f3**) and (**e4**)/(**f4**) are the corresponding true-color images, DEMs, and profiles of SMDI, DEM and black soil layer thickness, respectively.

Meanwhile, the strong correlations between *SMDI* and FC, as well as soil properties, confirmed that *SMDI* could reliably reflect SWRC. The spatial patterns in Figure 10 reveal key localities where soil hydraulic function has changed across the landscape. At Youyi Farm, the sandy plot (Figure 10b1–b4) exhibited a precipitous decline in *SMDI* (|*ΔSMDI*| > 0.15) where sand content exceeded 60%, indicating a critical threshold response to textural degradation. This cliff-like transition provides a quantitative criterion for rapid diagnosis of soil granularization. In Figure 10c1–c4, *SMDI* demonstrated a significant negative correlation with DEM, reflecting SOM depletion at slope shoulders induced by water erosion. The synchronous reduction in *SMDI* validates its sensitivity to erosion intensity. At Heshan Farm, terrain exerted pronounced control over *SMDI* spatial patterns (Figure 10e1–e4,f1–f4). On slope tops and steep gradients where black soil thickness is generally <40 cm (Figure 10e4,f4), *SMDI* values declined markedly, consistent with severe degradation. Conversely, slope foot and catchment positions exhibit high *SMDI* (40–60 cm soil depth), reflecting depositional accumulation of water-retentive materials. These findings demonstrated that *SMDI* effectively captures soil degradation patterns and rapidly identifies zones of compromised water retention capacity. Unlike conventional soil-quality assessments that rely predominantly on the static inversion of physicochemical properties, this framework advances the field by capturing dynamic hydraulic signatures. Consequently, these results can support precision irrigation management and hydrological modeling in agricultural systems, while providing critical soil moisture parameters for ecosystem dynamics and stability analyses.

### 4.3. Limitations and Broader Applicability Validation of SMDI

Although this study successfully established *SMDI* to characterize the SWRC and enable high-precision spatial monitoring, certain limitations remain to be addressed. Firstly, while the *SMDI* demonstrated robust performance in the spring agricultural landscapes of Heilongjiang Province, the current framework did not account for the impacts of straw incorporation on soil hydraulic properties, thereby potentially constraining its applicability in conservation tillage systems. Straw returning significantly enhances the soil organic carbon (SOC) content in the surface layer (0–20 cm) and improves water retention characteristics [45]. Future research should quantify straw–SWRC interactions to extend the index validity across diverse agricultural management regimes. Secondly, the *SMDI* developed in this study utilized a time window spanning from the onset of snowmelt to the bare-soil stage. While this window provided optimal vegetation-free conditions for optical remote sensing, *SMDI* becomes inoperable once crop canopies develop or when persistent cloud cover obstructs optical acquisition. Compared with microwave-based retrievals (e.g., SMAP, SMOS), which penetrate vegetation but suffer from coarse spatial resolution (>25 km), *SMDI* achieved field-scale 10 m resolution yet remains limited to bare-soil periods. Furthermore, the current results primarily characterize surface SWRC, with limited sensitivity to deep-profile moisture dynamics. Integrating our approach with SAR techniques [46] could potentially combine the spatial advantages of optical time series with all-weather penetration capabilities, enabling comprehensive monitoring across soil depths and growing seasons. Finally, to ensure broad applicability, *SMDI* requires validation across contrasting soil types, climatic regimes, and agricultural management systems to establish a robust scientific foundation for operational deployment in precision agriculture and ecosystem management. Additionally, clay mineralogy (e.g., swelling clays such as smectites and vermiculite versus non-swelling clays, silts, and sands) potentially affects water retention capacity, as swelling clays absorb water within their lattice structures [47], while non-swelling minerals do not. This study did not conduct X-ray diffraction (XRD) analysis to identify clay mineral types [48]. Future research should combine XRD analysis with hyperspectral visible-to-shortwave infrared (VSWIR) sensing to examine this mineralogical effect on SWRC.

## 5. Conclusions

This study constructed the *SMDI* to quantify SWRC through optical remote-sensing time series. The following conclusions were drawn: (1) Remote sensing signatures using Sentinel-2 time-series *NDWI* can successfully capture SWRC. The exponential decay pattern of the *NDWI* during the bare-soil window period after snowmelt effectively distinguished differences in water dynamics across various soil textures (e.g., clay showed the slowest decay, while sandy soil exhibited the fastest) and under different black soil layer thicknesses. (2) EDFM exhibited exceptional fit to the time-series *NDWI* (R^2^ = 0.84–0.99), enabling precise extraction of key physical parameters such as the attenuation rate (b) and stability level (c). This approach successfully transformed traditional point-scale static measurements into spatially continuous, non-destructive monitoring data. (3) The *SMDI* developed in this study showed high consistency (R = 0.82–0.88) with in situ sensor monitoring results and exhibited significant positive correlations with FC and SOM, demonstrating its ability to accurately reflect the SWRC governed by soil physical structure. (4) The *SMDI* spatial mapping clearly delineated how topography and erosion governed SWRC, accurately identifying impaired water-holding functions in severely eroded and desertified areas. This provides crucial digital decision-making support for precise black soil conservation, fertility restoration, and refined agricultural water management.

## Data Availability

The field measurement data and soil physical property data generated during this study are available from the corresponding author upon reasonable request.

## Supplementary Materials

The following supporting information can be downloaded at: https://www.mdpi.com/article/10.3390/s26123709/s1. Figure S1: Time-series NDWI across soil textures during snowmelt to bare-soil period; Figure S2: Time-series MNDWI across soil textures during snowmelt to bare-soil period; Figure S3: Time-series NDWI across slope positions during snowmelt to bare-soil period; Figure S4: Time-series MNDWI across slope positions during snowmelt to bare-soil period; Table S1: Bootstrap uncertainty quantification of EDFM parameters based on 2000 randomly selected pixels.

## Abbreviations

The following abbreviations are used in this manuscript:

DEM	Digital Elevation Model
EDFM	Exponential Decay Fitting Model
FC	Field Capacity
HD	Heavily Degraded
HS	High-Slope
LD	Lightly Degraded
LS	Low-Slope
MD	Moderately Degraded
MS	Moderate-Slope
ND	Non-Degraded
NEC	Northeast China
*NDWI*	Normalized Difference Water Index
*SMDI*	Soil Moisture Decay Index
SOM	Soil Organic Matter
SWRC	Soil Water Retention Capacity
SWRS	Soil Water Retention Signatures
*ΔSMDI*	Change in *SMDI*
