# Supplementary material for: Remote Sensing of Soil Water Retention Signatures Using Sentinel-2 Time-Series and Exponential Decay Fitting Model
Source: Sensors (Basel). 2026 Jun 10;26(12):3709. doi: 10.3390/s26123709 (PMC13306281; doi:10.3390/s26123709)
Supplement: Supplementary file 1 [file sensors-26-03709-s001.zip › sensors-4332699-supplementary.pdf]

Table S1. Bootstrap uncertainty quantification of EDFM parameters based on 2000 randomly selected pixels.

Parameter	Mean	Std	Median	P2.5	P97.5	Mean 95% CI half-width	Median 95% CI half-width
a	0.0713	0.0222	0.0692	0.0330	0.1205	0.0253	0.0130
b	12.6197	3.1830	13.0640	5.0478	17.5260	6.5322	6.5473
c	-0.0866	0.0495	-0.0729	-0.1829	-0.0174	0.0105	0.0100

Note: Mean, standard deviation (Std), and median describe the central tendency and dispersion of the 2000 pixel-level parameter medians. P2.5 and P97.5 are the 2.5th and 97.5th percentiles of these 2000 medians, constituting the 95% reference range of the parameter estimates. Mean and median 95% confidence interval (CI) half-widths quantify the uncertainty of the parameter estimate for an individual pixel, derived from 500 bootstrap resampling iterations. The median coefficient of determination (R2) of the EDFM fitting for these 2000 pixels is 0.908.

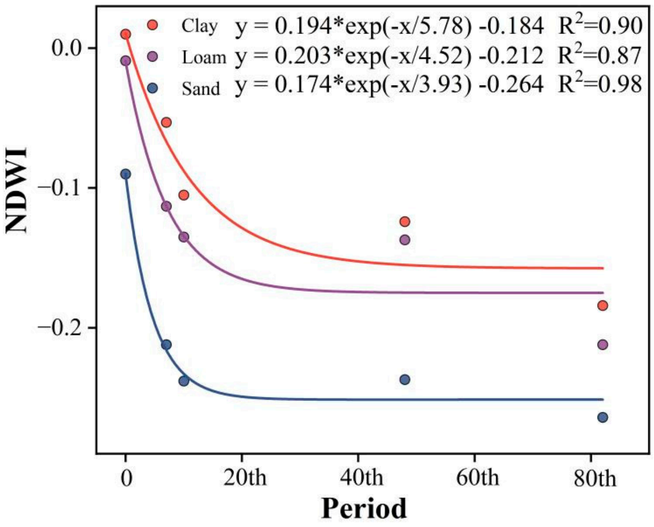

Figure S1. Time-series NDWI across soil textures during snowmelt to bare-soil period

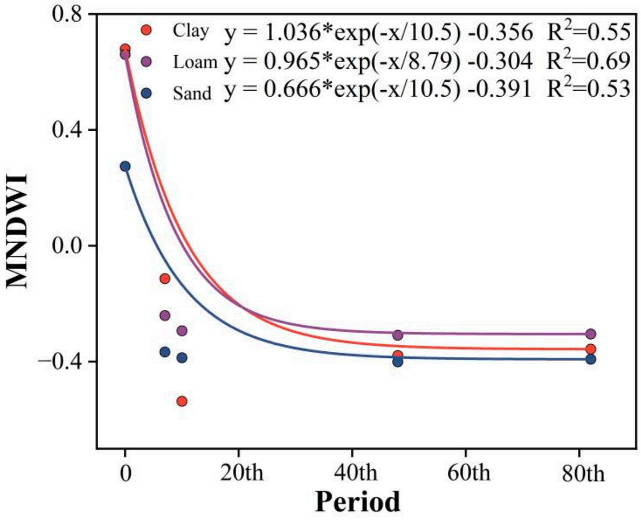

Figure S2. Time-series MNDWI across soil textures during snowmelt to bare-soil period

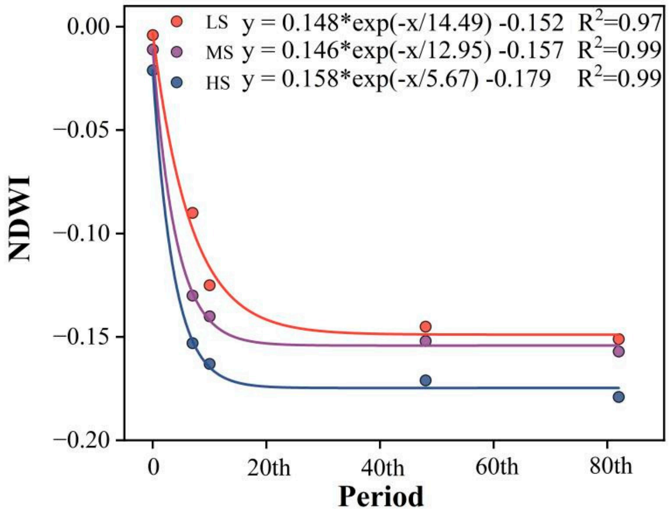

Figure S3. Time-series NDWI across slope positions during snowmelt to bare-soil period

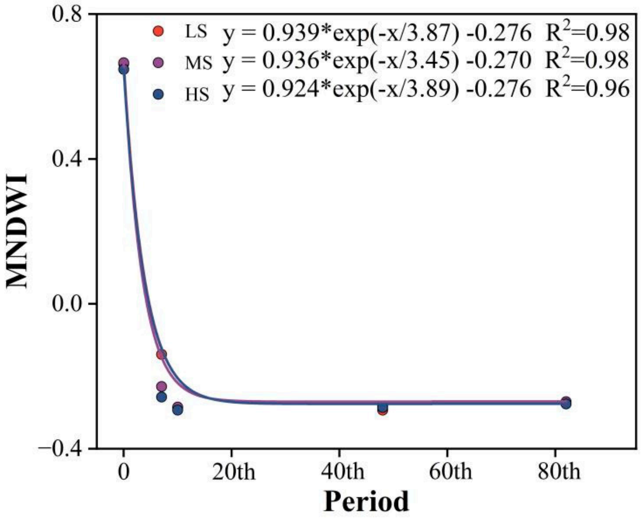

Figure S4. Time-series MNDWI across slope positions during snowmelt to bare-soil period
